# Glioblastoma Multiforme in the over 70's: “To treat or not to treat with radiotherapy?”

**DOI:** 10.1002/cam4.2398

**Published:** 2019-07-04

**Authors:** Aisling M. Glynn, Guhan Rangaswamy, Julianne O'Shea, Mary Dunne, Roger Grogan, Stephen MacNally, David Fitzpatrick, Clare Faul

**Affiliations:** ^1^ St Luke's Radiation Oncology Network Dublin Ireland; ^2^ Department of Neurosurgery Beaumont Hospital Dublin Ireland

**Keywords:** age, debulking, Glioblastoma, performance status, radiotherapy, Temozolomide

## Abstract

**Background:**

The incidence of Glioblastoma Multiforme (GBM) is increasing among the older population and is associated with poor prognosis. Management guidelines are lacking in this group. The purpose of this study was to analyze survival data and determine predictors of survival in patients aged ≥70 years treated with radiotherapy (RT) and/or Temozolomide.

**Materials and Methods:**

A retrospective analysis of all GBM patients treated at our institution between January 2011 and January 2017 was carried out.

**Results:**

One‐hundred and four patients were eligible. Median age was 73.8 years (70‐87). Thirty‐three patients received radical RT and 71 palliative RT. Overall median survival (MS) was 6 months. The MS was 10.6 months for radical patients and 4.9 months for palliative patients (*P *< 0.0005). The MS was 6.9 months in patients aged 70‐75 years and 5.2 months in those aged 76‐80 years (*P* = 0.004). The debulked group had a statistically significantly longer survival (8.0 months) than the biopsy only group (4.9 months). Biopsy only (hazard ratio [HR] 2.4), ECOG performance status 3 vs 0 (HR 6.4), and increasing age (HR 1.06) were associated with statistically significant shorter survival after adjustment for the effects of concurrent chemo, delay in starting RT, and RT dose.

**Conclusion:**

The MS for radical patients was favorable and approaching current literature for the under 70 age group. Radical treatment should be considered for good performance patients aged 70‐75 years. Increasing age was associated with shorter MS in patients aged ≥76 years. Debulking and good performance status were associated with improved survival.

## 1 INTRODUCTION

The incidence of Glioblastoma Multiforme (GBM) is increasing among the older population with approximately half of patients diagnosed with GBM aged 65 years or more.^1^ Older patients with GBM are expected to double by 2030.^2^ GBM is associated with a poor prognosis with a tendency toward limited intervention with advancing age.^1^ Median survival (MS) is approximately 4‐6 months.^1^, ^3^, ^4^, ^5^ While Radiotherapy (RT) has been the mainstay of management to date, older patients are underrepresented in trials and consensus guidelines on how to manage these patients are currently lacking. Additionally, classification of the “older'' patient varies in the literature from chronological age of “>65 years,” to “>70 years” or above, making comparison between trials and data interpretation difficult. The standard treatment of surgical resection and adjuvant chemotherapy and RT for patients with GBM is based on the trial by Stupp et al,^6^ which found a significant improvement in MS from 12.1 to 14.6 months with dual‐modality therapy compared to RT alone. However, this trial excluded patients aged greater than 70 years. The elderly are a unique and heterogeneous group and treatment options can be complicated by factors such as comorbidities, fragility, and increased susceptibility to treatment side effects. Therefore, a tailored treatment approach needs to be determined.

This study details our experiences of treating elderly patients, defined as 70 years of age or older, within our institution. Our objective was to analyze our survival data, to determine predictors of survival, and to propose an appropriate management plan for this patient cohort.

## 2 MATERIALS AND METHODS

Patient data were obtained from the national neuro‐oncology tertiary referral database and medical charts. Patients were included in this study if they had a histologically diagnosed GBM, treated with RT, with or without chemotherapy between January 2011 and January 2017. Treatment decisions were approved at the neuro‐oncology multidisciplinary meeting. Radical patients were defined as patients who received radical adjuvant RT of 60 Gray (Gy) in 30 fractions (#) as per the Stupp trial.^6^ Palliative patients were defined as patients treated with hypofractionated palliative RT regimens. Decisions regarding whether patients underwent radical or palliative RT schedules were determined by the joint decisions and expert opinions of the multidisciplinary team at the neuro‐oncology multidisciplinary team (MDT). Factors taken into consideration included patient comorbidities and polypharmacy, patient symptoms, neurological status, performance status, and extent of debulking. Detailed discussions took place on the potential benefits and toxicities associated with RT treatment. Patients with poor prognostic factors such as poor performance status, significant comorbidities, and/or minimally debulked were typically treated with palliative RT regimens.

Patients were excluded if they had radiological diagnosis alone, prior cranial irradiation, or if they were treated outside of our institution.

### 2.1 STATISTICAL ANALYSIS

Data were analyzed for overall survival which was the primary endpoint, measured from diagnosis (surgery/biopsy date) to death. The Kaplan‐Meier method was used to estimate survival times and the log‐rank test was used to compare differences in survival. A Cox proportional hazards model was used to assess the effects of covariates on survival. Categorical variables were analyzed using Chi‐squared tests. Analysis was done by IBM SPSS statistical software version 25. All statistical tests were two‐sided and assessed for significance at the 0.05 level.

## 3 RESULTS

**Figure 1:**
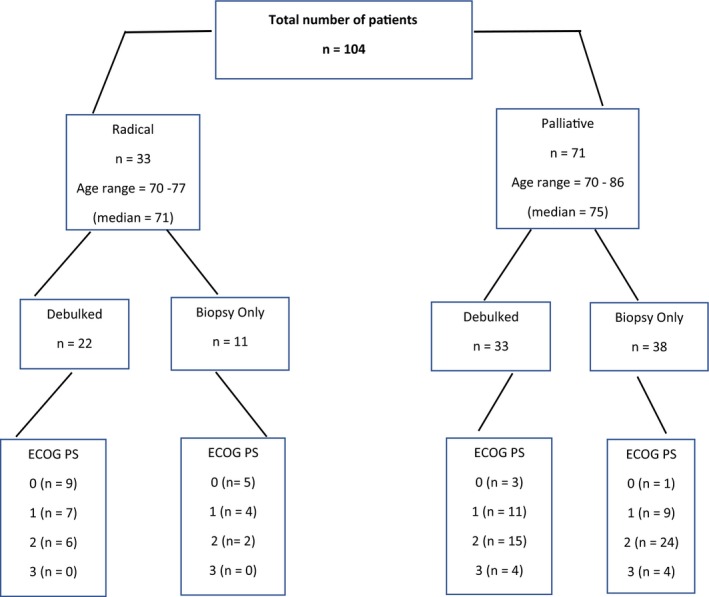
Overall patient characteristics by treatment intent, extent of surgery, and ECOG Performance Status (PS)

**Figure 2:**
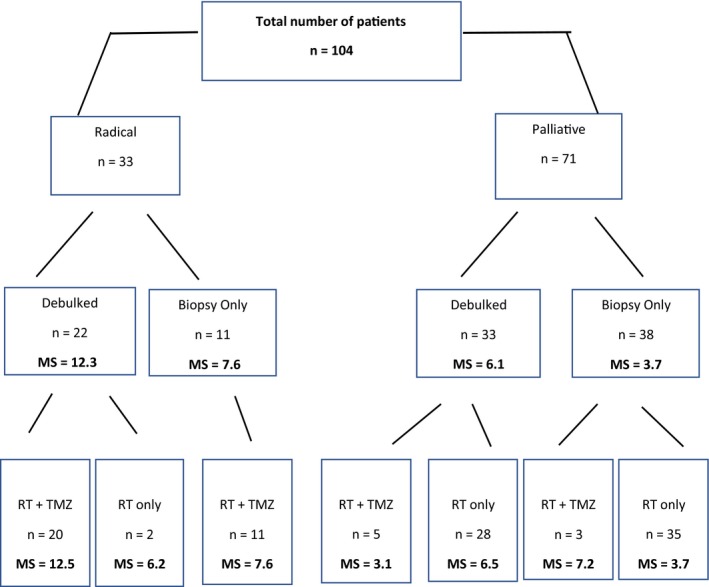
Overall treatment characteristics with median survival in months. RT, radiotherapy; TMZ, Temozolomide; MS, median survival

Of the 144 patients, older than the age of 70 years, diagnosed with GBM during the specified time period, 104 met the inclusion criteria. Table 1 summarizes the baseline patient characteristics for our patients. The median age at diagnosis was 73.8 years (range; 70‐87 years). Approximately, half (47%) had a performance status of 1 or better. Fifty‐three percent (n = 55) underwent debulking surgery and 47% (n = 49) had biopsy only. Twenty‐nine percent of patients were methylated and 35% were unmethylated. Thirty‐three patients underwent radical RT and 71 palliative RT. Figure 1 displays the patient characteristics, specifically performance status (PS) and surgical status, in each arm. All patients treated radically had a PS of ≤2. Figure 2 displays the MS for each group per treatment arm. The median radical dose delivered was 60 Gy and the median palliative dose was 38 Gy. A statistically significant association was found between age group and whether the patient was treated with radical or palliative intent. Forty‐six percent of patients aged 70‐75 years had radical treatment compared to 3% of those aged ≥76 years, χ^2^ (1, n = 104) = 17.4, *P* < 0.0005.

**Table 1:** Baseline demographic and patient characteristics

Characteristic	Patients (n = 104)	Percentage (%)
Age		
70‐75	70	67
76‐80	25	24
>81	9	9
Sex		
Male	63	61
Female	41	39
ECOG performance status		
0	18	17
1	31	30
2	47	45
3	8	8
Extent of surgical resection		
Biopsy only	49	47
Debulked	55	53
MGMT status		
Methylated	30	29
Unmethylated	36	35
Data not available	38	36

Abbreviations: ECOG, Eastern Cooperative Oncology Group; MGMT, methylguanine‐DNA methyltransferase.

The MS of the entire group was 6 months (95% CI of 5.1‐6.8 months), with a MS of 10.6 months for patients treated radically and 4.9 months for patients treated palliatively (*P *< 0.0005; Table 2). Survival was statistically significantly different by age group (*P* = 0.004); patients aged 70‐75 years had an overall MS of 6.9 months, those aged 76‐80 years had a MS of 5.2 months, and those aged >81 years had a MS of 4.6 months (Table 2).

**Table 2:** Median Survival by age group and treatment intent

Age group	Total Number	Total Estimate	Radical Number	Radical Estimate	Palliative Number	Palliative Estimate
		(95% CI)		(95% CI)		(95% CI)
70‐75	70	6.9 (5.7‐8.0)	32	10.6 (7.9‐13.3)	38	4.5 (2.9‐6.1)
76‐80	25	5.2 (3.1‐7.2)	1	3.4 (.‐.)	24	5.2 (3.5‐6.9)
81+	9	4.6 (0.0‐12.0)	0	—	9	4.6 (0.0‐12.0)
Overall	104	6.0 (5.1‐6.8)	33	10.6 (7.6‐13.6)	71	4.9 (3.8‐5.9)

Abbreviations: CI, Confidence Interval.

Radical patients who had RT and Temozolomide (TMZ) had a MS of 10.6 months compared to 6.2 months in those who had RT alone (*P* = 0.059). Radical patients who received TMZ and underwent debulking had a statistically significant longer MS time (12.5 months 95% CI: 8.2‐16.8) than those who had biopsy only (7.6 months 95% CI 5.7‐9.6; *P* = 0.014). There was no statistically significant difference in MS for palliative patients treated with RT & TMZ (4.1 months) compared to those treated with RT alone (5.0 months); *P* = 0.805. All patients who underwent debulking followed by RT & TMZ (n = 39) had a MS of 12.3 months while patients who had biopsy only followed by RT & TMZ had a MS of 7.3 months (*P* = 0.033) (Table 3). For those with MGMT status data, overall survival was not statistically significantly different between methylated and unmethylated patients.

**Table 3:** Overall Survival of Patients treated with RT & concurrent chemotherapy

	N	Median Estimate	95% Confidence Interval		*P*‐value
			Lower Bound	Upper Bound	
Radical RT					
Biopsy plus concurrent chemo	11	7.6	5.7	9.6	0.014
Debulked plus concurrent chemo	20	12.5	8.2	16.8	
Total	31	10.6	8.0	13.2	
Radical + Palliative RT					
Biopsy plus concurrent chemo	14	7.3	6.4	8.2	0.033
Debulked plus concurrent chemo	25	12.3	10.8	13.9	
Total	39	10.5	7.8	13.3	

Abbreviations: N, Number; *P*, probability value.

**Figure 3:**
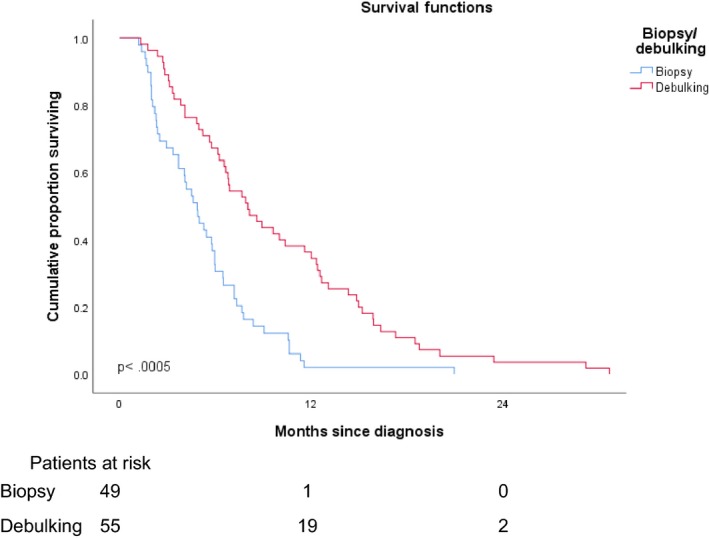
Median survival according to extent of surgery

**Figure 4:**
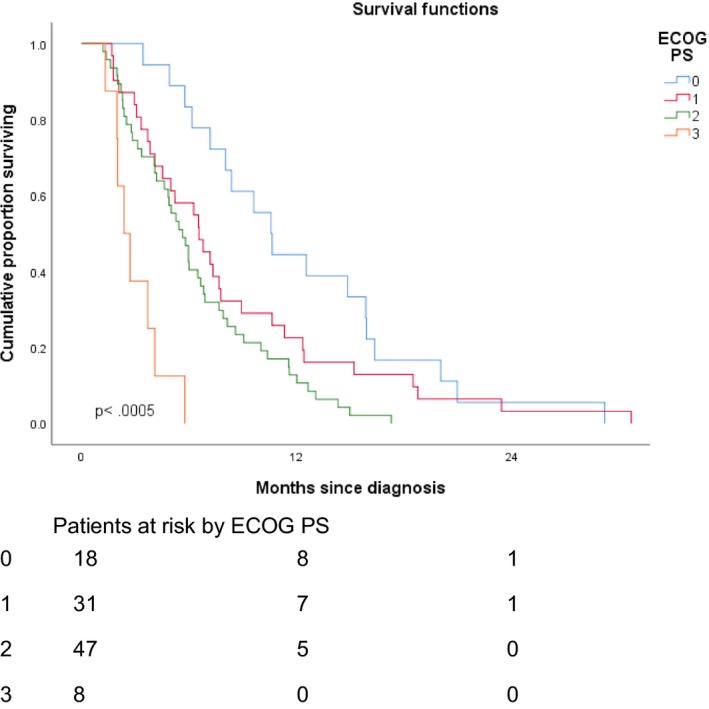
Median survival according to ECOG Performance status. ECOG, Eastern Cooperative Oncology Group

The debulked group had a statistically significantly longer survival time than the biopsy only group (Figure 3). The patients who underwent debulking had a MS of 8.0 months (95% CI, 5.8‐10.2 months) compared to 4.9 months in the biopsy only group (95% CI, 3.7‐6.0 months, *P *< 0.0005). A log‐rank test for trend showed statistically significantly longer survival times for those with better ECOG PS (*P *< 0.0005) (Figure 4). MS was 10.5, 6.5, 5.6, and 2.4 months, respectively, for those with ECOG PS of 0, 1, 2, and 3.

In Cox regression modeling, gender was not predictive of survival. Independent predictors of survival included age at diagnosis, ECOG PS, and the extent of surgery, as shown in Table 4. The risk of death was higher for older patients. The estimated hazard increases by 1.06 times for each increase of 1 year of age. Delay in starting RT, RT dose, and concurrent chemo was not predictive of survival. The strongest predictor of poorer outcome was ECOG PS = 3. The estimated hazard or risk of death increases 6.4 times in this group compared to the ECOG PS = 0 group, after adjustment for the effects of age, the extent of surgery, RT dose, concurrent chemo, and delay in starting RT (Hazard Ratio [HR] for death, 6.4; 95% CI, 2.3 to 17.6; *P* < 0.0005). The impact of performance status on survival by group (biopsy/debulking and radical/palliative treatment) could not be assessed due to the small patient numbers. Patients who had biopsy only had a shorter survival than those debulked. The estimated hazard or risk of death increases 2.4 times in this group compared to the debulked group, after adjustment for the effects of age, ECOG PS, RT dose, concurrent chemo, and delay in starting RT (HR for death, 2.4; 95% CI, 1.5 to 3.7; *P* < 0.005).

**Table 4:** Cox regression analysis on whole cohort of patients (n = 104)

	B	Sig.	HR	95.0% CI for HR	
				Lower	Upper
Age at diagnosis	0.060	0.046	1.062	1.001	1.127
ECOG PS		0.004			
ECOG PS 1 vs 0	0.420	0.194	1.522	0.807	2.869
ECOG PS 2 vs 0	0.699	0.033	2.013	1.059	3.824
ECOG PS 3 vs 0	1.851	0.000	6.367	2.308	17.562
Biopsy vs Debulked	0.870	0.000	2.386	1.522	3.743
No Concurrent chemo vs Concurrent chemo	0.431	0.259	1.539	0.728	3.254
Delay in starting RT vs No Delay	0.242	0.320	1.274	0.791	2.054
RT dose	−0.007	0.698	0.993	0.956	1.030

Abbreviations: B, Beta Coefficient; CI, Confidence Interval; HR, Hazard Ratio; Sig, Significance.

A separate cox regression analysis of the radical group was carried out. Independent predictors of survival included age and the extent of surgery, as shown in Table 5. Patients with biopsy only had shorter survival than those debulked. The estimated hazard or risk of death increases 4.5 times in those who had biopsy only compared to those who were debulked, after adjustment for the effects of age, ECOG PS, and RT dose (HR for death, 4.5; 95% CI, 1.7 to 11.4; *P* = 0.002). Additionally, the risk of death was higher for older patients. The estimated hazard increases by 1.4 times for each increase of 1 year of age, after adjustment for the effects of ECOG PS, the extent of surgery, and RT dose. ECOG PS and RT dose were not predictive of survival.

**Table 5:** Cox regression analysis on radical cohort of patients (n = 33)

	B	Sig.	Exp (B)	95.0% CI for Exp (B)	
				Lower	Upper
Age at diagnosis	0.306	0.028	1.357	1.033	1.784
ECOG PS		0.097			
ECOG PS (1)	0.378	0.387	1.459	0.620	3.436
ECOG PS (2)	1.080	0.031	2.946	1.104	7.859
Biopsy/Debulked	1.495	0.002	4.461	1.739	11.443
RT dose	−0.049	0.550	0.952	0.809	1.119

Abbreviations: B, Beta Coefficient; CI, Confidence Interval; Exp (B), Exponentiation; Sig, Significance.

## 4 DISCUSSION

Our goal was to assess survival rates for the older patients with GBM, to analyze predictors of survival, and provide guidance on how these patients should be managed. The age cutoff for “older'' patients remains controversial given the heterogeneous performance status within this patient cohort. We defined older patients as those aged ≥70 years. This age was chosen as we have an aging population in whom the incidence of GBM is likely to increase. Additionally, the Stupp et al landmark trial^6^ excluded patients greater than 70 years of age and hence a “standard approach'' does not exist for this population group.

Our overall MS of 6 months is consistent with previous studies.^3^, ^7^ When analyzed in terms of treatment intent, we found patients aged 70‐75 years treated radically with standard RT and concomitant TMZ had a favorable outcome with a MS of 10.6 months. Radical patients who received TMZ and underwent debulking had a MS time of 12.5 months. These figures compare well with the Stupp protocol results.^6^

Our results are in concordance with previously reported trials in patients ≥70 years. In one Italian prospective trial, 32 patients, aged ≥70 years, who underwent surgery followed by adjuvant RT (60 Gy/30#) with concomitant and adjuvant TMZ were found to have a MS of 10.6 months and the median progression‐free survival was 7 months.^8^ Chang‐Halpenny et al^9^ carried out a retrospective review of 129 elderly patients with a median age of 70 years. Patients treated with standard RT and concomitant TMZ had a median time to death of 13 months compared to 5.4 months in the patients treated with abbreviated RT and TMZ. In the Chang‐Halpenny et al review, the “older” elderly patient (median age 75 years) and the patient with fewer gross total resections and lower karnofsky performance score (KPS) tended to receive abbreviated RT,^9^ similar characteristics to the patients receiving palliative RT in our cohort.

More recently, hypofractionated RT regimens with concomitant TMZ have been suggested as treatment options for elderly GBM patients with some promising results.^10^ However, our overall MS of 10.6 months in our radical cohort receiving concurrent TMZ exceeds the MS of 9.3 months seen in the Perry et al study.^10^ The median age of the patients in the Perry et al study was similar to ours at 73 years. They reported a MS of 9.3 months in the hypofractionated RT (40.05 Gy in 15 fractions) and TMZ group compared to 7.6 months in the RT alone group. Of note, the percentage of surgical resections, 68.3% in the Perry et al study and the 67% in our radical group were similar, and KPS in both ranged from 0 to 2.

Our patients treated with “palliative” RT regimens did not do well regardless of age (MS of 4.9 months). Roa et al compared survival in older patients receiving abbreviated RT (40 Gy/15#) versus standard RT (60 Gy/30#) and found similar MS of 5.6 months versus 5.1 months.^7^ Unlike Roa et al, we found a statistically significant survival difference between those treated with standard RT compared to palliative RT regimens (*P *< 0.0005). It needs to be acknowledged that while our study found a poor overall MS for patients treated with palliative RT, there may be a role for shorter course RT in suitable patients^10^, ^11^ especially in patients unlikely to tolerate a standard RT regimen and the hospital/outpatient commitment attached to this schedule. Malmstrom et al conducted a study focusing at TMZ alone, versus standard RT versus hypofractionation (34 Gy/10#) RT. For age older than 70 years, survival was better with TMZ and with hypofractionated RT than with standard RT. HR for hypofractionated versus standard RT was 0.59 (95% CI 0.37‐0.93), *P* = 0.02.^11^ Of note, only patients with PS 0‐2 were included in this study (except a score of 3 owing to neurological deficit), while our palliative cohort had 11% of patients with PS = 3. Neurological toxicity and steroid use have been found to be less in hypofractionated RT compared to standard RT,^12^ something not evaluated in our study.

The role of TMZ as monotherapy is another consideration for elderly patients, not explored in our current study. TMZ given to tumor MGMT promoter methylation has been associated with longer survival than those without MGMT promoter methylation.^11^ Of note, Malmstrom et al found no difference in survival between those methylated or unmethylated treated with RT. We also found no difference in outcome based on MGMT status. However, MGMT status was unavailable in 36% of our patients and only 29% were methylated.

Biopsy only, ECOG PS 3 vs 0, and increasing age were all found to be independent predictors of poor outcome in our cohort of patient. We found the strongest predictor of poorer outcome was ECOG PS = 3. The estimated hazard or risk of death increases 6.4 times in this group compared to the ECOG PS = 0 group. Our radical group, who had the better overall MS was found to only have patients with PS 0‐2, with 76% of patients having a PS ≤ 1. Previous studies exploring the role of RT in elderly GBM patients, with promising findings, have been carried out in good PS patients. Keime‐Guibert et al compared RT (50 Gy/1.8 Gy per fraction) and best supportive care (BSC) versus BSC alone, found a significantly improved MS of 7 months compared to 4 months in the BSC alone group. All patients had a KPS of 70 or more.^13^ These studies show that the patients who benefit from RT are those with a good performance status. Similar results to ours were found by Harris et al^14^ who evaluated elderly patients (aged ≥ 75 years) to determine survival outcomes following intensity modulated radiotherapy. On univariate analysis, the independent predictors of survival included younger age (*P* = 0.02), better performance status (*P* = 0.014), and greater resection extent (*P* = 0.002), additionally they found TMZ use (*P*<0.001) to be predictive of survival. In a recursive partitioning analysis estimation, Scott et al^15^ identified four prognostic subgroups based on the extent of surgery, age (>/<75), and KPS score (>/<70) in three cohorts of GBM patients > 70 years and found decreasing MS in patients with reduced performance status and in those who had limited surgical intervention. Poor KPS is consistently found to be a poor prognostic factor in the survival of elderly GBM patients^14^, ^16^, ^17^, ^18^ and remains an important factor when determining treatment decisions, in addition to age.

Our analysis showed that advancing age was associated with poor survival. MS for patients aged ≥76 years was 4.8 months. The estimated hazard or risk of death increased by 1.06 times for each increase of 1 year of age. After adjustment for the effects of other covariates, there would be an estimated 10.6‐fold increase in HR for death of a patient aged 80 years compared to a patient aged 70 years. When analyzed separately, age was also found to be one of two significant predictive factors associated with better outcome in the radical group (extent of surgery being the other predictive factor). The estimated hazard increased by 1.4 times for each increase of 1 year of age, after adjustment for the effects of ECOG PS, the extent of surgery, and RT dose. Our results are consistent with previous literature citing older age as a significant factor of poor survival.^1^, ^15^ A previous population‐based study found no difference in survival between younger and older patients treated with surgery alone or BSC suggesting that lower survival rates in older patients with GBM may be in part due to a lesser response to RT.^1^ Quality of survival decreases with advancing age^19^ and patients not fit for radical therapy should ideally be spared the possible toxicities of a treatment that potentially offers little survival advantage. Our study found a statistically significant association between age and treatment intent with only 3% of patients aged 76 years or older treated radically. The median age of our radically treated group was 71 years. A population‐based review by Iwamoto et al^4^ found that age was the most significant predictor of resection, RT, or chemotherapy; with advancing age associated with decreasing use of all three modalities.

Consistent with the literature, our cohort of patients who had biopsy only, had a poorer outcome (4.9 months vs 8.0 months in the debulked group). Biopsy only patients were 2.4 times more likely to have an event (HR 2.4). Noorbakhsh et al^20^ reported a 2 to 3 month improvement in overall survival in patients undergoing gross total resection in comparison to subtotal resection across all ages. Chang‐Halpenny et al^9^ found that more extensive surgery was associated with longer survival time (HR 0.466). Kita et al^1^ reported age was not found to be predictive of poor survival in patients treated with surgery alone, highlighting the fact that surgical resection remains a valid treatment option in carefully selected patients who may not receive further adjuvant treatment. A meta‐analysis and systematic review carried out by Almenawer et al 2015, which included patients aged ≥60 years, 34 studies, and 12607 participants, found that when tumor resection (of any extent) was compared to biopsy only, there was a mean difference of 3.88 months in OS (95% CI: 2.14‐5.62, *P* < 0.001) postoperative KPS, progression free survival, and mortality were also found to be superior in the resection patients.^21^ Based on our findings and previous studies, tumor resection, when deemed safe for the patient, should always be considered in the management of the elderly GBM patient.

There are some limitations to our study related to its retrospective nature. Decisions to treat radically or palliatively were based on MDT outcomes. Hence, the better outcome in radical patients may be related to the selection of better prognosis patients. Moving forward, the involvement of a oncogeriatrican and standardized evaluation tools should also be incorporated into MDT to help guide treatment decisions.^22^, ^23^

Quality of life, which is especially important in GBM, was not always available from the notes and hence not evaluated in this study. Neurotoxicity and side effects of RT were not evaluated. Very few of our palliative cohort treated with abbreviated RT regimens, received concomitant TMZ. Despite this, our study includes a large cohort of patients from a single institution, with a strict definition of elderly age, evaluating treatment practices, and survival in this challenging age group. The survival rate of our patients treated with Stupp protocol^6^ in the 70‐75 years age group, especially when surgically debulked, approaches the results of the Stupp trial,^6^ which only included patients aged ≤70 years.

With our aging population, we are seeing a new generation of elderly patients who are fit and active and we believe, based on our encouraging survival rates for patients aged 70‐75 years treated with Stupp protocol,^6^ that we now have a “young” elderly category and that the term “elderly” for GBM patients should be re‐defined as age ≥76 years. Combined modality treatment with standard dose RT should be considered for carefully selected patients ≤75 years. This recommendation is supported by a review by Laperriere et al who recommended that patients aged ≥70 years with good PS (WHO PS 0‐2) and good resection could be considered for standard RT & TMZ therapy.^24^ Above the age of 76 years, unfortunately, we found patients treated with RT had a reduced survival. We found age is not just a number and cannot be ignored. Treatment decisions for elderly GBM patients should take into consideration age, extent of surgery, and PS.

## 5 CONCLUSION

Our study showed patients aged 70‐75 years with newly diagnosed GBM and treated radically had survival rates comparable with younger age groups. Treatment of patients in this age group with standard 60 Gy in 30 fractions and concomitant TMZ is a valid treatment approach. Patients undergoing palliative RT had less favorable outcomes. Increasing age was associated with poorer outcome and reduced survival with a significantly reduced overall survival in patients aged 76 years or greater. Further research in patients aged over 76 years is required to determine if these patients should be spared the toxicities associated with RT. This cohort of patients may be better managed with BSC alone. In concordance with previous research, age, surgical debulking, and good performance status were independent predictors of improved survival.

Taking into consideration this diverse patient group, we have recommended the following management approach in our institution:

1. All ages—maximal surgical resection if feasible
2. Age 70‐75 years
  - Debulked, good performance status—standard approach radical RT/TMZ
  - Biopsy only, good performance status—standard approach radical RT/TMZ versus short course RT (±TMZ)
  - Poor performance status—discuss short course RT (±TMZ) versus BSC
3. Age ≥ 76 years
  - Debulked, good performance status—discuss short course RT (±TMZ) versus BSC
  - Biopsy only, poor performance status—BSC

## CONFLICT OF INTEREST

None.

## AUTHOR CONTRIBUTION

AMG conceptualization, data curation, formal analysis, investigation, methodology, project administration, resources, software, supervision, validation, visualization, writing—original draft, and writing—review and editing. GR conceptualization, data curation, formal analysis, investigation, methodology, project administration, resources, software, supervision, validation, visualization, writing—review and editing. JO' conceptualization, data curation, formal analysis, investigation, methodology, resources, software, supervision, validation, visualization. MD formal analysis, investigation, methodology. RG data curation, resources, software. SM data curation, resources. DF conceptualization, investigation, methodology. CF conceptualization, methodology, project administration, resources, supervision, validation, visualization, writing ‐ review and editing.
